# Comparison of Volatile Compounds of Some Medicinal Plants from Lamiaceae Family by HS-SPME Method

**DOI:** 10.3390/ijms27104601

**Published:** 2026-05-20

**Authors:** Zeynep Ergun, Elmira Ziya Motalebipour, Nesibe Ebru Kafkas, Mujgan Guney

**Affiliations:** 1 Department of Biology, Faculty of Engineering and Natural Sciences, Osmaniye Korkut Ata University, Osmaniye 80000, Turkey; zeynepergun@osmaniye.edu.tr; 2 Department of Agronomy and Plant Breeding, Faculty of Agriculture, Water, Food and Nutraceuticals, Isf. C, Islamic Azad University, Isfahan 1477893780, Iran; e.ziyamotalebipour@iau.ac.ir; 3 Medicinal Plant Research Center, Isf. C., Islamic Azad University, Isfahan 1477893780, Iran; 4 Department of Horticulture, Faculty of Agriculture, Cukurova University, Adana 01330, Turkey; ebruyasakafkas@gmail.com; 5 Department of Horticulture, Faculty of Agriculture, Yozgat Bozok University, Yozgat 66900, Turkey

**Keywords:** plant biodiversity, medicinal plant, bioactive compounds, HS-SPME, GC-MS

## Abstract

This study investigates the volatile composition of twelve medicinal plant species belonging to the Lamiaceae family, which are widely recognized for their diverse biological activities, including antioxidant, antibacterial, and antifungal properties. Despite extensive studies on essential oils, comparative analyses using solvent-free techniques under different microclimatic conditions remain limited. This study investigates the volatile compounds in twelve medicinal plants from the Lamiaceae family using headspace solid-phase microextraction coupled with gas chromatography–mass spectrometry (HS-SPME/GC-MS). Lamiaceae plants are recognized for their diverse medicinal properties, including antioxidative, antibacterial, and antifungal effects. A total of 74 volatile compounds were identified, encompassing terpenes, alcohols, esters, aldehydes, and ketones. Notably, *Lavandula spica* L. exhibited the highest number of unique volatiles (28), while *Melissa officinalis* L. had the fewest (16). Key compounds included Citral (65.48%) in *Melissa officinalis* L., Menthol (33.37%) and *Menthyl acetate* (30.53%) in *Mentha piperita* L., Carvone (45.86%) in *Mentha spicata* L., and Eucalyptol (54.71%) in *Origanum syriacum* L. Plants from Adana Botanic Park were rich in terpenes and ketones, whereas those from Osmaniye contained higher levels of alcohols, aldehydes, and esters. The findings emphasize the impact of geographic location on volatile profiles and suggest avenues for further research into medicinal efficacy and optimal dosage. This study supports the sustainable use of plant biodiversity (SDG 15) and highlights the importance of bioactive compounds for human health and well-being (SDG 3).

## 1. Introduction

The Lamiaceae family, comprising 236 genera and 6900 to 7200 species, stands out as one of the most significant herbal families due to its diverse array of medicinal plants [1]. Among the prominent genera with notable medicinal potential are *Lavandula*, *Mentha*, *Melissa*, *Origanum*, *Rosmarinus*, *Salvia*, *Sideritis*, and *Thymus* [2]. Lavenders (*Lavandula* spp.), a standout member of the Lamiaceae family, include more than 39 species distributed across Arabia, the Mediterranean region, Asia, Northern Africa, and the Middle East [3].

*Lavandula* species are characterized by aromatic compounds such as linalool, α-pinene, limonene, cineole, cis- and trans-ocimene, 3-octanone, camphor, caryophyllene, and terpinene-4-ol [4]. *Lavandula spica* L. and *Lavandula dentata* L., which are endemic and widely distributed in the Mediterranean part of Turkey, hold special importance. Lavender is in high demand in flavoring, food, and pharmaceutical industries due to its robust aroma [5]. A study indicated that the use of lavender oil aromatherapy among individuals diagnosed with fibromyalgia led to a significant enhancement in their quality of life [6].

The *Mentha* genus, comprising 18 species, is another famous source of medicinal herbs within the Lamiaceae family. This genus is widely distributed in North America, Europe, Asia, and Africa. Mints are valued for their anti-inflammatory and analgesic properties, making them a valuable tool in folk medicine for addressing various ailments. Among the main species within this genus are *Mentha spicata* and *Mentha piperita* L. *Mentha piperita* L. is a hybrid of *M. spicata* and *M. aquatic*. The key components extracted from *Mentha* are identified as menthol, Menthone, and 1,8 cineol [7].

The *Melissa* genus stands out as an aromatic herb within the Lamiaceae family, with its native range encompassing Europe, Asia, southwestern Siberia, and northern Africa. Notably, *Melissa officinalis* L., known as lemon balm, English balm, and sweet balm, is another member of the mint family. Key compounds present in *M. officinalis* L. include citronellol, citral, and linalool [8]. These compounds contribute to its antiviral and antibacterial effects, making it a valuable treatment for ailments caused by viruses and bacteria.

The genus *Origanum* is a subshrub within the Lamiaceae family, native to Europe, Asia, and North Africa. This genus is pivotal in traditional medicine, particularly in addressing respiratory complaints. With over 18 hybrids and 43 species, *Origanum* holds significant diversity [9].

*Origanum majorana* L., *Origanum syriacum* var. *bevanii*, and *Origanum syriacum* L. are endemic species, widely distributed in the Mediterranean region. Notable compounds found in the *Origanum* genus include terpinene-4-ol, cis-sabinene, and o-cymene, with their quantities varying among different species [10].

*Rosmarinus officinalis* L., commonly known as rosemary, is a perennial herbaceous plant renowned for its antioxidant and anti-inflammatory properties, which also belongs to the Lamiaceae family and is native to the Mediterranean region. However, due to its valuable culinary and medicinal attributes, it has been widely cultivated across the globe [3]. The versatility of *Rosmarinus officinalis* L. (rosemary) is evident through its extensive use in the realms of cooking, medicine, and cosmetics. Its robust flavor and aromatic qualities have contributed to its popularity as a culinary ingredient, while its therapeutic potential has been acknowledged for centuries [3]. Beyond its culinary and medicinal roles, rosemary’s antibacterial and antifungal attributes have led to its inclusion in aromatherapy, where it is believed to aid in stress reduction and memory enhancement. The compound makeup of rosemary is notably rich in terpenes, including alpha-pinene, beta-pinene, and camphor. These constituents contribute to its distinct properties and applications [11].

*Salvia* L., commonly referred to as sage, is a notable genus within the mint family, Lamiaceae. This genus encompasses a diverse array of plants, exceeding 900 species that are distributed globally, primarily in temperate regions. Among the prominent members of the *Salvia* genus, *Salvia officinalis* L. holds particular significance. This species boasts a broad spectrum of primary and secondary metabolites, contributing to its valuable medicinal and nutritional attributes. Secondary metabolites in *Salvia officialis* L. include terpenoids, phenolic acids, flavonoids, and tannins. Notably, terpenoids such as thujone, camphor, and cineole are present, each possessing distinct medicinal properties. Thujone, for instance, has been linked to various effects, while camphor and cineole are recognized for their antimicrobial and antifungal properties. These compounds collectively contribute to the diverse therapeutic potential associated with sage [12].

*Sideritis* spp., commonly referred to as mountain tea, stands as a noteworthy genus of flowering plants within the Lamiaceae family. Indigenous to the Mediterranean region, this genus has gained popularity as an herbal tea. With a substantial presence of over 150 species, *Sideritis* has a rich history in traditional medicine due to its recognized anti-inflammatory, antioxidant, and antimicrobial attributes. In more recent times, scientific exploration has illuminated the potential of *Sideritis* as a treatment for Alzheimer’s disease and other neurological disorders. This research has underscored its significance beyond traditional applications [13]. The secondary metabolites found within *Sideritis* spp., notably rosmarinic acid and apigenin, are particularly noteworthy for their significant antioxidant and anti-inflammatory properties. These compounds contribute to the diverse therapeutic potential of Sideritis, making it a valued herbal remedy with potential implications for various health conditions [14].

*Thymus* L., a perennial shrub belonging to the Lamiaceae family, is more commonly recognized as thyme. Originating in the Mediterranean region, this plant has been cultivated and valued for its aromatic and medicinal attributes over many centuries. Among the various species within this genus, *Thymus vulgaris* L. stands out, renowned for its remarkable medicinal qualities. This species has earned a prominent place as a natural remedy, addressing an array of health concerns, including respiratory infections, digestive troubles, and inflammatory conditions. The therapeutic potential of *Thymus vulgaris* L. can be attributed to its composition, which is rich in compounds like thymol and carvacrol. These bioactive constituents contribute to the plant’s antibacterial, antiviral, and antifungal properties. The presence of these compounds underscores its traditional and contemporary uses as a powerful and versatile herbal remedy [15].

Indeed, the medicinal plant species within the Lamiaceae family hold considerable significance in natural medicine, pharmacology, cosmetology, and aromatherapy due to their diverse secondary metabolites. These include alcohols, aldehydes, ketones, esters, terpenes, and phenolic compounds, which confer antimicrobial, antiviral, antioxidant, and anti-inflammatory activities [16,17,18].

Despite extensive studies on essential oils of Lamiaceae species obtained mainly through hydrodistillation, little is known about their volatile composition when analyzed using solvent-free techniques that preserve thermolabile and low-volatility compounds. Moreover, the influence of distinct microclimates on the volatile profiles of these medicinal plants remains underexplored.

Therefore, the novelty of this study lies in applying HS-SPME/GC-MS to comparatively profile the volatiles of 12 medicinal plants collected from two different Turkish provinces (Adana and Osmaniye), thereby addressing the gap in knowledge on how geographical origin affects volatile composition when assessed by this technique.

## 2. Result and Discussion

### 2.1. Volatile Compounds of the Lamiaceae Family

**Figure 1:**
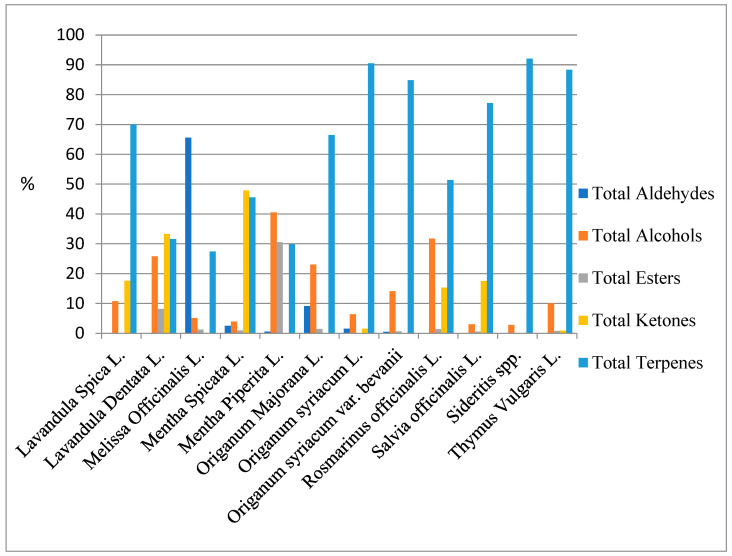
Total aroma compounds (%) by chemical family in 12 species of Lamiacea family.

Table 1 includes information on the percentage component distribution, retention time, and retention indices for each volatile compound identified in the studied samples. Detected volatile compounds within the Lamiaceae family encompass a diverse array of chemical family groups, comprising 3 volatiles in aldehydes, 23 volatiles in alcohols, 7 volatiles in esters, 3 volatiles in ketones, and 37 volatiles in terpenes. Out of the 74 volatile compounds investigated in this study, terpenes exhibited the widest diversity, while the smallest range was observed in aldehydes (Figure 1). The presence of volatile compounds varied in terms of abundance across different species, ranging from high to low quantities. Additionally, certain compounds were not detected in certain studied species. Terpenes were found to have the highest percentage of volatiles, whereas esters exhibited the lowest percentage. Among the volatile compounds, terpenes emerged as the dominant category for the Lamiaceae family species.

The investigation encompassing 12 distinct Lamiaceae species yielded diverse volatile compound profiles. Specifically, *Lavandula spica* L. featured 28 volatiles, while *Lavandula dentata* L. and *Mentha spicata* each contained 23 volatiles. *Mentha piperita* L. exhibited 20 volatiles, *Melissa officinalis* L. had 16, and *Origanum majorana* L. had 25. Notably, *Origanum syriacum* var. *bevanii* showcased 22, while *Origanum syriacum* L. and *Rosmarinus officinalis* L. each featured 21 and 26 volatiles, respectively. *Salvia officinalis* L. contained 21 volatiles, *Sideritis* spp. L. had 18, and *Thymus vulgaris* L. featured 24. The most extensive and least extensive species-specific volatile components were observed in *Lavandula spica* L. with 28 volatiles and *Melissa officinalis* L. with 16 volatiles, respectively (Table 1).

In various species of the Lamiaceae family, the terpenes category exhibited notable high-quantity compounds such as eucalyptol, p-cymene, α-thujone, trans-sabinene hydrate, caryophyllene, and β-Pinene. Among the alcohol chemical family, l-(-)-menthol, α-terpineol, 1-Borneol, and Linalool were found in significant quantities. Notably, the esters category was characterized by high quantities of Menthyl acetate and neryl acetate. In terms of aldehydes, citral emerged as a high-quantity compound. Lastly, carvone and camphor stood out as compounds of substantial quantity within the ketones category (Table 2).

This comprehensive study underscores the diverse range of volatile compounds within different Lamiaceae species. Significant percentages of volatile compounds were identified in specific species. For instance, citral from the Aldehyde chemical family was notably present in *Melissa officinalis* L. at a percentage of 65.48%. Among the Alcohol chemical family, L(-)Menthol was detected in *Mentha piperita* L. at a level of 33.37%. Menthyl acetate, categorized within the Ester chemical family, stood out in *Mentha piperita* L. at a level of 30.53%. Within the Ketone chemical family, carvone was identified in *Mentha spicata* L. at a percentage of 45.86%. Noteworthy, eucalyptol, a member of the Terpen chemical family, was found in *Origanum syriacum* L. at a percentage of 54.71% (Table 2).

**Figure 2:**
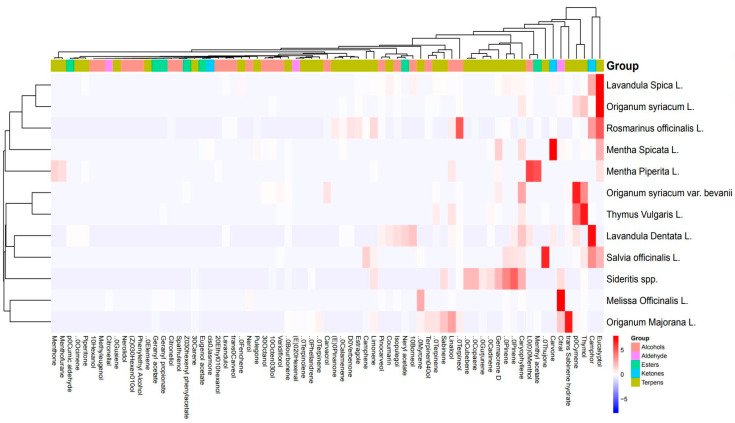
Heatmap of volatile aroma profiles in 12 Lamiaceae species obtained by HS-SPME, illustrating the distribution and relative abundance of major chemical compounds across taxa.

The heatmap analysis for the aroma profile of 12 species from the Lamiaceae family reveals distinct groupings of species based on their concentrations of specific chemical compounds such as alcohols, aldehydes, esters, ketones, and terpenes (Figure 2). Each species exhibits a unique chemical composition that contributes to its aroma, with several notable patterns emerging from the analysis. *Mentha* species, including *Mentha spicata* L. and *Mentha piperita* L., are particularly rich in menthol, menthone, and other related compounds, placing them in a distinct cluster due to their strong minty aroma profiles. On the other hand, *Melissa officinalis* L. stands out with high levels of citral, making it unique among the other species for its lemon-like scent. The presence of high aldehyde concentrations in *Melissa officinalis* L. contrasts with species like *Lavandula spica* L. and *Lavandula dentata* L., which are rich in linalool and geranyl acetate, compounds that contribute to their sweet and floral scents commonly associated with lavender oils.

**Figure 3:**
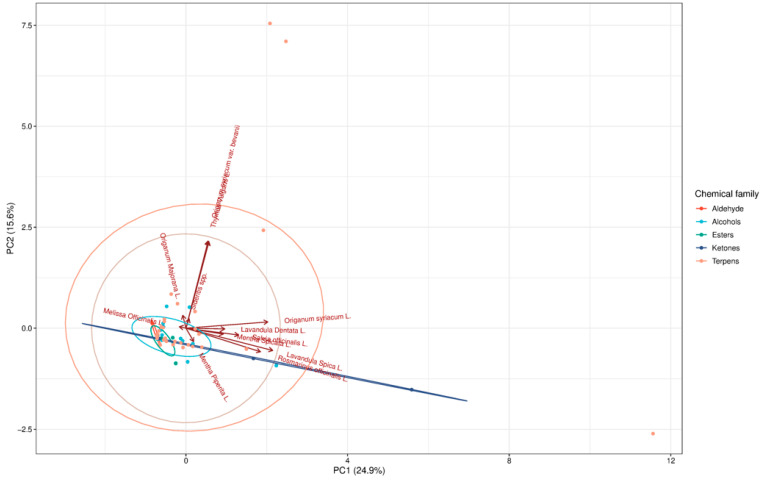
Principal component analysis (PCA) of volatile aroma profiles in 12 Lamiaceae species obtained by HS-SPME, showing separation of taxa according to major chemical families.

The principal component analysis (PCA) also clearly separated the studied Lamiaceae species based on their volatile compound profiles obtained by HS-SPME. The first two principal components (PC1: 24.9%, PC2: 16.5%) together explained over 40% of the total variance, indicating that a considerable proportion of the chemical variation among species was captured. Samples such as *Origanum syriacum* and *Lavandula stoechas* were distinctly positioned along PC1, largely influenced by high contributions from terpenes and ketones, while *Melissa officinalis* and *Mentha spicata* clustered closer together, reflecting their higher alcohol contents. The strong loadings of compounds such as linalool, geraniol, and camphor suggest that these metabolites were the main drivers of separation among species. The PCA biplot therefore highlights the chemical diversity of Lamiaceae volatiles and demonstrates that terpenes and alcohols are the most discriminative chemical families. This chemometric approach provides a clear comparative framework, enabling differentiation of closely related taxa based on their volatile fingerprints (Figure 3).

Terpenes such as thymol, carvacrol, and eucalyptol are prominent in species like *Thymus vulgaris* L., *Origanum syriacum* L., and *Rosmarinus officinalis* L., which group together due to their strong herbal and camphoraceous aromas, making them valuable in medicinal and antiseptic applications. The dendrogram included in the heatmap illustrates these relationships by clustering species that share similar concentrations of these key compounds. For example, the close clustering of *Origanum majorana* L. and *Origanum syriacum* L. reflects their similar terpene profiles, while species like *Sideritis* spp. and *Salvia officinalis* L. form another cluster based on their diverse chemical makeup, particularly in terms of their high terpene content.

The clustering of species based on their chemical profiles helps to identify potential uses for each plant. For instance, species high in alcohols like Linalool (e.g., lavender species) are ideal for perfumery, while those rich in menthol and menthone (e.g., mint species) are suitable for medicinal and culinary purposes. The high terpene concentrations in species like *Thymus vulgaris* L. and *Origanum syriacum* L. highlight their antimicrobial and antifungal properties, making them valuable for therapeutic use. Overall, this heatmap analysis underscores the chemical diversity within the Lamiaceae family and provides a comprehensive framework for understanding how different species can be clustered based on their aroma profiles for various applications.

### 2.2. Variation in Volatile Compounds Among Plant Species

The analysis of aromatic compounds within the Lamiaceae family has unveiled a diverse range of constituents that significantly contribute to their distinct scent profiles and potential therapeutic attributes. Within *Lavandula spica* L., Eucalyptol emerges as a prominent element, constituting a significant portion (41.29%) alongside Camphor (15.21%). Eucalyptol carries a refreshing, camphor-like fragrance with a hint of peppery coolness. Its alluring aromatic and flavor qualities make it valuable in fragrances and cosmetics and as a flavor enhancer. Eucalyptol has demonstrated antinociceptive properties, suggesting potential calming and central nervous system depressant effects [19]. Inhalation of eucalyptus aromatherapy oil, abundant in over 80% of eucalyptol, has proven effective in mitigating post-COVID-19 syndrome symptoms, such as breathlessness, back pain, and anxiety affecting individuals worldwide and potentially bearing labor and financial repercussions [6]. Furthermore, the therapeutic potential of eucalyptol spans cardiovascular treatments, antimicrobial effects, anti-inflammatory benefits, and respiratory disorder support, corroborated by numerous studies [1]. Conversely, Eucalyptol remains undetected in *Lavandula dentata* L., where Camphor (29.82%), Caryophyllene (8.58%), 1-Borneol (8.4%), and Neryl acetate (6.37%) emerge as primary volatile compounds. The prevalence of camphor-rich compounds in *Lavandula* species is validated by multiple scientific publications [4]. However, some researchers have reported linalool as the principal compound in the essential oil of *Lavandula* species [20]. Both Camphor and 1-Borneol have demonstrated extensive and diverse pharmacological effects, encompassing anti-inflammatory, analgesic, and antipyretic properties and notable antimicrobial activity [21].

*Melissa officinalis* L. exhibited a notable abundance of Citral (65.48%) and β-Myrcene (23.06%). Previous studies have documented the presence of Citral in *Melissa* species [22], while the occurrence of β-Myrcene was initially observed in this study. Myrcene finds application in cosmetics, soaps, detergents, and as a versatile food additive. It also holds a foundational role in creating essential fragrances such as menthol, citral, citronellol, and others, which are pivotal in the production of commercially significant compounds. Several studies have also revealed the properties of β-Myrcene, encompassing anxiolytic, antioxidant, anti-aging, anti-inflammatory, and analgesic attributes [23].

The dominant volatile compounds in *Mentha spicata* L. were Carvone (45.86%) and eucalyptol (16.27%). On the other hand, the primary volatile compounds in *Mentha piperita* L. consisted of L-(-)-Menthol (33.37%), Menthyl acetate (30.53%), Menthone (8.9%) and Eucalyptol (6.31%).

The investigation by Park et al. [24] reported eucalyptol (46.28 ± 4.25), caryophyllene (5.47 ± 0.49), and menthol (3.02 ± 0.22) as the major components in *Mentha* spp., which are quite similar to the volatile compounds identified in the present study, although caryophyllene was found at a higher level in our results.

In the current study, the highlighted volatile compounds of three *Origanum* species: *Origanum majorana* L., *Origanum syriacum* L., and *Origanum syriacum* var. bevanii comprised trans sabinene hydrate (30.63%), eucalyptol (54.71%), and p-cymene (36.21%), respectively. Terpenes have consistently emerged as the key active components in Oregano species [25]. *Rosmarinus officinalis* L. exhibited α-Terpineol (22.19%), camphor (13.92%), and eucalyptol (22.42%) as its primary compounds. This study marks the first identification of α-Terpineol and eucalyptol in this context. *Salvia officinalis* L. featured α-thujone (30.97%) and camphor (17.54%) as its prominent volatile compounds. In the case of *Sideritis* spp., the major component was β-pinene (18.09%), differing from the observations of Kardali et al. [26] which α-pinene was the primary compound. *Thymus vulgaris* L. demonstrated richness in thymol (42.43%) and p-cymene (27.6%).

The species of the Lamiaceae family for this study were collected from two distinct locations, Adana Botanik Park and Osmaniye, providing insight into the contrasting climate of these regions. The discernible differences in weather patterns and climatic attributes reflect the distinctive conditions between Osmaniye and Adana. Osmaniye in southern Turkey exhibits a Mediterranean climate characterized by hot, dry summers and moderate, wet winters. Meanwhile, Adana, located nearby, shares a Mediterranean climate but tends to be slightly warmer and more arid due to its lower elevation and proximity to the Mediterranean Sea. This results in Adana often having warmer summertime temperatures and receiving less precipitation compared to Osmaniye. Despite the overall similarity in climate, these subtle variations in temperature and rainfall underscore the existence of microclimatic nuances, even within close proximity. These factors may contribute to the observed variations in volatile compounds; however, differences between species should also be considered. According to the literature, variations in volatile compounds are influenced by factors such as genetic background, location, seasonal changes, and developmental stages [27,28]. For instance, environmental conditions, including climate and edaphic factors, have been shown to significantly affect volatile composition and chemotype variability in *Origanum vulgare* populations [29]. Similarly, climatic parameters such as precipitation and geographic origin were reported to influence key volatile compounds in *Mentha pulegium* populations collected from different habitats [30]. Therefore, although species-related differences are predominant, the possible contribution of microclimatic conditions to the observed volatile profiles cannot be excluded.

## 3. Materials and Methods

### 3.1. Material

Leaves of twelve distinct members of the Lamiaceae plant family, including Sweet Marjoram, Lemon Balm, Bible Hyssop, Hybrid Mint, Greek Sage, Lavender, Fringed Lavender, Za’atar, Spearmint, Rosemary, Sage, and Thyme, without undergoing any treatments, were used in the current study. The plant materials were collected from Ali Nihat Gokyigit Botanical Garden (*n* = 7) and Osmaniye (n = 5) in July 2019 (Table 3), corresponding to the transition between vegetative growth and early flowering stage. Formal taxonomic identification of the plant material was performed by Dr. Zeynep Ergun based on regional floristic references and morphological characteristics. A voucher specimen of this material was not deposited in a publicly accessible herbarium. Following collection, the plant samples were appropriately labeled based on their origin and were subsequently stored at a temperature of −20 °C. For subsequent analyses, one gram of each ground plant material was used.

### 3.2. Methods

#### 3.2.1. HS-SPME Extraction of Volatile Compounds

Volatile compounds were extracted using the Headspace-Solid Phase Microextraction (HS-SPME) technique. Ground samples were homogenized with 5 M calcium chloride (CaCl_2_) to increase ionic strength and facilitate the release of volatile compounds into the headspace, as described in similar extraction protocols [31]. A 100 µm PDMS fiber was employed because it is widely used for monoterpene-rich matrices such as Lamiaceae essential oils. Although CAR/PDMS fibers were also tested, the PDMS fiber yielded a higher number of compounds in our samples and therefore was selected for subsequent analyses. The fiber was exposed to the sample headspace and incubated for 30 min at 30 °C with magnetic stirring. The extraction time was chosen based on preliminary trials showing equilibrium after 30 min, consistent with the literature [31].

#### 3.2.2. GC/MS Analysis

Analysis was conducted using a Shimadzu GC/MS equipped with a DB-Wax column (30 m × 0.25 mm × 0.5 µm). Helium was used as the carrier gas at a flow rate of 1.4 mL/min (adjusted to the optimal linear velocity for this column). The oven temperature program started at 40 °C, increased at 10 °C/min to 200 °C (16 min), and was held for 10 min, giving a total run time of ~26 min. This correction addresses a discrepancy in the original text regarding ramp duration. Compound identification was performed tentatively by comparing mass spectra with NIST/Wiley libraries and retention indices. Retention indices were calculated against a C7–C30 n-alkane series under identical conditions, and reference RI values for DB-Wax were obtained from the NIST database. Quantification was expressed as a relative percentage of total ion current (TIC) peak areas, which provides a semi-quantitative comparison among samples but does not account for compound-specific ionization efficiencies. All analyses were performed in triplicate. Relative standard deviations (RSD%) were calculated for major compounds to assess analytical precision.

#### 3.2.3. Statistical Analysis

Multivariate analyses were performed using R software version 4.4.1. Principal component analysis (PCA) was conducted to evaluate variation among samples based on volatile compound profiles. In addition, heatmap analysis was applied to visualize clustering patterns and the relative abundance of compounds across samples. Hierarchical clustering was performed using Euclidean distance and the complete linkage method. Data were standardized (row-wise scaling) prior to analysis, and heatmaps were generated using the pheatmap package in R version 4.4.1 (2024-06-14).

## 4. Conclusions

The Lamiaceae family includes numerous species widely used in traditional medicine. In this study, twelve species collected from Adana and Osmaniye (Turkey) exhibited diverse volatile profiles comprising aldehydes, alcohols, esters, ketones, and terpenes. Among these, terpenes were the dominant chemical group across all species, consistent with previous essential oil studies. Unlike many earlier works that applied hydrodistillation, our study utilized HS-SPME/GC-MS, which allowed detection of thermolabile and low-volatility compounds that are often lost during conventional extraction. This represents one of the first comparative HS-SPME-based surveys of multiple Lamiaceae species grown under different microclimatic conditions. The comparative results demonstrated that plants collected from Osmaniye were relatively richer in aldehydes, alcohols, and esters, whereas plants from Adana contained higher levels of ketones and terpenes. These findings highlight the significant influence of local microclimatic factors on volatile composition, an aspect rarely considered in previous research. It is important to note that the compounds were tentatively identified based on library matching and retention indices, and quantification was semi-quantitative. Therefore, results should be interpreted as relative rather than absolute concentrations. Future work should validate major compounds using pure standards, apply mixed-phase SPME fibers for broader coverage, and integrate chemometric tools (PCA, cluster analysis) to better resolve interspecies differences. Overall, this study contributes new insights into the volatile diversity of Lamiaceae species under different Turkish microclimates and demonstrates the utility of HS-SPME/GC-MS as a complementary approach to hydrodistillation. These results may support future standardization of medicinal plant products and guide cultivation strategies aimed at optimizing the yield of bioactive volatile constituents.

## Figures and Tables

**Table 1:** Number of Aroma Compounds per Chemical Family in 12 Species of Lamiacea Family.

NO	Chemical Family/Species	Aldehydes	Alcohols	Esters	Ketones	Terpenes
1	*Lavandula spica* L.	0	8	0	2	18
2	*Lavandula dentata* L.	0	7	2	2	12
3	*Melissa officinalis* L.	2	6	2	0	6
4	*Mentha spicata* L.	1	8	2	3	9
5	*Mentha piperita* L.	1	6	1	0	12
6	*Origanum majorana* L.	2	4	3	0	16
7	*Origanum syriacum* var. *bevanii*	1	8	1	1	10
8	*Origanum syriacum* L.	1	9	1	0	11
9	*Rosmarinus officinalis* L.	0	9	1	2	14
10	*Salvia officinalis* L.	0	5	1	1	14
11	*Sideritis* spp.	0	3	0	0	14
12	*Thymus vulgaris* L.	0	9	2	1	12

**Table 2:** Volatile compounds, retention times, retention indices, and percentage compositions of the studied species of the Lamiaceae family.

Chemical Family	Compounds Name	R.T	RI	DB-wax	*1*	*2*	*3*	*4*	*5*	*6*	*7*	*8*	*9*	*10*	*11*	*12*
**Aldehyde**	(E)-2-Hexenal	10.05	1228	1227	N.D.	N.D.	N.D.	N.D.	N.D.	0.8	N.D.	0.48	N.D.	N.D.	N.D.	N.D.
	Citronellal	17.40	1490	1488	N.D.	N.D.	0.11	N.D.	N.D.	N.D.	N.D.	N.D.	N.D.	N.D.	N.D.	N.D.
	Citral	23.75	1742	1741	N.D.	N.D.	65.48	2.47	0.54	8.29	1.52	N.D.	N.D.	N.D.	5.2	N.D.
	Total Aldehydes				N.D.	N.D.	65.59	2.47	0.54	8.29	1.52	N.D.	N.D.	N.D.	N.D.	N.D.
**Alcohols**	1-Hexanol	13.69	1356	1359	N.D.	N.D.	N.D.	N.D.	N.D.	N.D.	N.D.	N.D.	N.D.	N.D.	N.D.	N.D.
	(Z)-3-Hexen-1-ol	14.60	1388	1379	N.D.	N.D.	N.D.	N.D.	N.D.	N.D.	0.26	N.D.	N.D.	N.D.	N.D.	N.D.
	3-Octanol	14.86	1396	1393	N.D.	N.D.	N.D.	0.96	0.12	N.D.	N.D.	1.6	N.D.	N.D.	N.D.	N.D.
	1-Octen-3-ol	16.49	1458	1450	N.D.	N.D.	N.D.	1.74	N.D.	N.D.	0.36	1.84	N.D.	0.08	0.6	0.39
	2-Ethyl-1-hexanol	17.54	1495	1495	N.D.	N.D.	N.D.	N.D.	1.42	N.D.	N.D.	N.D.	N.D.	N.D.	N.D.	N.D.
	Linalool	19.12	1556	1556	0.79	2.21	1	0.33	5.12	13.91	0.11	0.19	3.88	0.06	N.D.	6.44
	Isopulegol	19.50	1571	1571	1.46	5.53	0.12	N.D.	N.D.	N.D.	1.38	N.D.	0.43	N.D.	N.D.	N.D.
	Terpinen-4-ol	20.45	1607	1602	N.D.	N.D.	0.06	0.23	N.D.	5.1	0.43	N.D.	0.97	N.D.	N.D.	N.D.
	L-(-)-Menthol	21.42	1647	1645	0.76	3.95	N.D.	N.D.	33.37	0.36	1.92	0.19	N.D.	N.D.	N.D.	0.26
	Pinocarveol	21.78	1661	1654	1.95	3.13	0.18	N.D.	N.D.	N.D.	0.36	0.54	1.08	0.38	0.21	0.33
	α-Terpineol	22.80	1701	1706	1.16	1.69	N.D.	N.D.	N.D.	3.65	1.57	0.89	22.19	1.5	1.95	1.07
	1-Borneol	22.93	1708	1706	3.7	8.4	N.D.	N.D.	N.D.	N.D.	N.D.	N.D.	N.D.	N.D.	N.D.	N.D.
	Lavandulol	24.30	1760	1686	0.61	0.88	N.D.	N.D.	N.D.	N.D.	N.D.	N.D.	0.99	N.D.	N.D.	N.D.
	Citronellol	24.51	1774	1764	N.D.	N.D.	0.6	N.D.	N.D.	N.D.	N.D.	N.D.	0.51	N.D.	N.D.	N.D.
	Nerol	25.28	1807	1797	N.D.	N.D.	3.12	0.15	N.D.	N.D.	N.D.	N.D.	N.D.	N.D.	N.D.	0.3
	trans-Carveol	26.03	1841	1833	N.D.	N.D.	N.D.	N.D.	N.D.	N.D.	N.D.	N.D.	1.11	N.D.	N.D.	N.D.
	Phenylethyl Alcohol	27.83	1921	1918	0.3	N.D.	N.D.	0.18	N.D.	N.D.	N.D.	N.D.	N.D.	N.D.	N.D.	N.D.
	Methyleugenol	30.13	2029	2030	N.D.	N.D.	N.D.	N.D.	N.D.	N.D.	N.D.	N.D.	N.D.	N.D.	N.D.	N.D.
	Nerolidol	30.53	2049	2014	N.D.	N.D.	N.D.	0.14	N.D.	N.D.	N.D.	N.D.	N.D.	N.D.	N.D.	N.D.
	Carvacrol	34.03	2224	2240	N.D.	N.D.	N.D.	0.18	0.35	N.D.	N.D.	6.09	0.56	N.D.	N.D.	0.32
	Veridiflorol	31.46	2093	2092	N.D.	N.D.	N.D.	N.D.	0.12	N.D.	N.D.	2.45	N.D.	1.02	N.D.	0.6
	Spathulenol	35.73	2306	2129	N.D.	N.D.	N.D.	N.D.	N.D.	N.D.	N.D.	0.27	N.D.	N.D.	N.D.	0.3
	Total Alcohols				10.73	25.79	5.08	1.21	38.96	23.02	5.77	10.62	31.72	0	0	0
**Esters**	Menthyl acetate	18.69	1540	1541	N.D.	N.D.	N.D.	0.2	30.53	0.88	0.12	N.D.	1.34	0.54	N.D.	N.D.
	Neryl acetate	23.64	1738	1737	N.D.	6.37	N.D.	N.D.	N.D.	0.17	N.D.	N.D.	N.D.	N.D.	N.D.	0.16
	Geranyl acetate	24.36	1768	1768	N.D.	N.D.	0.4	N.D.	N.D.	0.4	N.D.	N.D.	N.D.	N.D.	N.D.	N.D.
	p-Cumic aldehyde	24.90	1791	1794	N.D.	1.79	N.D.	N.D.	N.D.	N.D.	N.D.	N.D.	N.D.	N.D.	N.D.	N.D.
	Geranyl propionate	25.78	1829	1828	N.D.	N.D.	0.83	N.D.	N.D.	N.D.	N.D.	N.D.	N.D.	N.D.	N.D.	0.61
	Eugenol acetate	33.17	2181	2263	N.D.	N.D.	N.D.	0.75	N.D.	N.D.	N.D.	N.D.	N.D.	N.D.	N.D.	N.D.
	Z-3-hexenyl phenylacetate	34.32	2238	2220	N.D.	N.D.	N.D.	N.D.	N.D.	N.D.	N.D.	0.65	N.D.	N.D.	N.D.	N.D.
	Total Esters				0	8.16	1.23	0.75	0	0.57	0	0.65	0	0	0	0
**Ketones**	Camphor	18.20	1520	1536	15.21	29.82	N.D.	0.54	N.D.	N.D.	1.49	N.D.	13.92	17.54	N.D.	0.87
	Carvone	23.86	1740	1744	2.38	3.44	N.D.	45.86	N.D.	N.D.	N.D.	N.D.	1.38	N.D.	N.D.	N.D.
	cis-Jasmone	28.13	1935	1938	N.D.	N.D.	N.D.	1.46	N.D.	N.D.	N.D.	N.D.	N.D.	N.D.	N.D.	N.D.
	Total Ketones				17.59	33.26	0	47.86	0	0	1.49	0	15.3	0	0	0
**Terpens**	α-Pinene	4.98	1006	1015	3.31	N.D.	0.82	N.D.	N.D.	1.36	N.D.	0.19	1.69	6.15	14.6	N.D.
	Camphene	5.85	1068	1059	1.3	N.D.	N.D.	N.D.	N.D.	0.29	N.D.	N.D.	1.69	7.94	N.D.	N.D.
	β-Pinene	6.71	1102	1096	2.6	3.54	N.D.	N.D.	0.5	0.7	N.D.	N.D.	N.D.	5.13	18.09	N.D.
	Sabinene	7.05	1117	1119	1.54	N.D.	N.D.	N.D.	0.19	9.41	N.D.	N.D.	N.D.	0.14	4.88	N.D.
	α-Phellandrene	8.23	1162	1160	N.D.	N.D.	N.D.	N.D.	N.D.	1.44	N.D.	N.D.	N.D.	N.D.	0.96	N.D.
	β-Myrcene	8.34	1166	1178	N.D.	N.D.	23.06	N.D.	0.54	3.33	N.D.	N.D.	N.D.	1.64	N.D.	N.D.
	α-Terpinene	8.98	1188	1183	N.D.	N.D.	N.D.	N.D.	N.D.	3.06	N.D.	N.D.	N.D.	N.D.	N.D.	N.D.
	Limonene	9.20	1195	1193	1.79	N.D.	N.D.	N.D.	1.34	3.38	N.D.	N.D.	6.29	2.88	4.59	N.D.
	Eucalyptol	9.53	1207	1202	41.29	N.D.	N.D.	16.27	6.31	N.D.	54.71	N.D.	22.42	11.57	N.D.	N.D.
	γ-Terpinene	10.64	1249	1251	2.15	0.71	N.D.	N.D.	N.D.	5.3	1.23	2.2	N.D.	0.16	N.D.	4.6
	3-Carene	10.93	1259	1162	N.D.	N.D.	0.58	N.D.	N.D.	0.25	N.D.	N.D.	N.D.	N.D.	0.56	N.D.
	p-Cymene	11.43	1276	1275	0.45	4.83	N.D.	N.D.	N.D.	2.4	9.88	36.21	0.9	0.7	N.D.	27.6
	α-Terpinolene	11.75	1287	1288	N.D.	N.D.	N.D.	N.D.	N.D.	1.34	N.D.	N.D.	N.D.	0.18	N.D.	N.D.
	α-Fenchene	14.94	1399	1402	2.29	N.D.	N.D.	N.D.	N.D.	N.D.	N.D.	N.D.	N.D.	N.D.	N.D.	N.D.
	α-Thujone	15.63	1425	1416	N.D.	N.D.	N.D.	N.D.	N.D.	N.D.	N.D.	N.D.	N.D.	30.97	N.D.	0.83
	α-Cubebene	16.61	1462	1460	N.D.	N.D.	N.D.	N.D.	N.D.	N.D.	N.D.	N.D.	N.D.	N.D.	8.87	N.D.
	Menthone	16.78	1492	1473	N.D.	N.D.	N.D.	N.D.	8.9	N.D.	N.D.	N.D.	N.D.	N.D.	N.D.	N.D.
	trans Sabinene hydrate	16.79	1468	1474	1.24	2.02	N.D.	1.85	N.D.	30.63	0.53	2.45	N.D.	0.59	N.D.	1.08
	Menthofurane	17.46	1492	1497	N.D.	N.D.	N.D.	N.D.	7.35	N.D.	N.D.	N.D.	N.D.	N.D.	N.D.	N.D.
	α-Copaene	17.64	1498	1491	N.D.	N.D.	N.D.	N.D.	N.D.	N.D.	N.D.	0.51	0.28	N.D.	9.08	0.23
	β-Bourbonene	18.36	1527	1528	N.D.	1.22	N.D.	0.68	0.59	1.67	N.D.	2.2	N.D.	N.D.	0.63	0.85
	α-Gurjunene	18.68	1539	1529	N.D.	N.D.	N.D.	N.D.	N.D.	0.63	N.D.	N.D.	N.D.	N.D.	3.76	N.D.
	(E)-Pinanone	19.00	1552	1548	N.D.	N.D.	N.D.	N.D.	0.2	N.D.	N.D.	N.D.	3.56	N.D.	N.D.	N.D.
	β-Ocimene	20.14	1594	1233	0.55	1.96	N.D.	N.D.	N.D.	N.D.	0.12	N.D.	N.D.	N.D.	N.D.	N.D.
	β-Elemene	20.24	1598	1599	N.D.	N.D.	N.D.	N.D.	0.41	N.D.	N.D.	N.D.	N.D.	N.D.	N.D.	N.D.
	β-Guaiene	20.26	1599	1663	N.D.	N.D.	0.06	N.D.	N.D.	N.D.	N.D.	N.D.	N.D.	N.D.	N.D.	N.D.
	Caryophyllene	20.37	1603	1608	2.78	8.58	2.54	8.75	N.D.	N.D.	6.72	15.88	0.7	4.62	9.82	5.65
	D-Verbenone	22.98	1709	1723	0.27	1.1	N.D.	N.D.	N.D.	N.D.	N.D.	N.D.	4.94	N.D.	N.D.	N.D.
	Germacrene D	23.23	1720	1710	1.94	N.D.	N.D.	10.65	2.77	N.D.	0.31	4.35	0.78	N.D.	9.49	1.22
	Piperitone	23.55	1734	1735	0.87	1.64	N.D.	N.D.	0.7	N.D.	N.D.	N.D.	N.D.	N.D.	N.D.	0.39
	σ-Cadinene	24.39	1769	1749	N.D.	0.85	N.D.	0.64	N.D.	N.D.	N.D.	0.25	N.D.	N.D.	5.08	3.07
	α-Calamenene	26.12	1845	1837	N.D.	0.8	N.D.	1.66	N.D.	N.D.	0.41	N.D.	2.61	N.D.	N.D.	0.39
	Pulegone	26.63	1867	1631	N.D.	N.D.	N.D.	2.75	N.D.	N.D.	N.D.	N.D.	0.49	N.D.	N.D.	N.D.
	Thymol	33.52	2198	2146	1.41	N.D.	0.35	2.29	N.D.	1.27	14.12	20.24	0.64	4.52	1.65	42.43
	Estragole	22.38	1685	1685	0.99	N.D.	N.D.	N.D.	N.D.	N.D.	2.46	0.34	4.34	N.D.	N.D.	N.D.
	Coumarin	39.95	2459	2458	3.22	4.35	N.D.	N.D.	N.D.	N.D.	N.D.	N.D.	N.D.	N.D.	N.D.	N.D.
	Total Terpens				69.99	31.6	27.41	45.54	29.8	66.46	90.49	84.82	51.33	77.19	92.06	88.34

**Table 3:** Studied twelve different species of the Lamiaceae family.

No	Name	Species	Place of Collection
1	Lavender	*Lavandula spica* L.	Adana Botanik Park
2	Fringed Lavender or French Lavender,	*Lavandula dentata* L.	Adana Botanik Park
3	Lemon Balm	*Melissa officinalis* L.	Adana Botanik Park
4	Spearmint, Garden Mint, Common Mint, Lamb Mint, Mackerel Mint	*Mentha spicata* L.	Osmaniye
5	Hybrid Mint (Peppermint)	*Mentha piperita* L.	Osmaniye
6	Marjoram	*Origanum majorana* L.	Adana Botanik Park
7	Bible Hyssop, Biblical-Hyssop, Lebanese Oregano, Syrian Oregano	*Origanum syriacum* var. *bevanii*	Adana Botanik Park
8	Za’atar	*Origanum syriacum* L.	Adana Botanik Park
9	Rosemary	*Rosmarinus officinalis* L.	Osmaniye
10	Common Sage, Sage	*Salvia officinalis* L.	Osmaniye
11	Greek Sage	*Sideritis* spp.	Adana Botanik Park
12	Common Thyme, German Thyme, Garden Thyme, Thyme	*Thymus vulgaris* L.	Osmaniye

## Data Availability

The original contributions presented in this study are included in the article. Further inquiries can be directed to the corresponding author.

## Abbreviations

The following abbreviations are used in this manuscript:

HS-SPME	Headspace Solid-Phase Microextraction
GC-MS	Gas Chromatography–Mass Spectrometry
*M.*	*Mentha*
TIC	Total Ion Current
RSD%	Relative Standard Deviation (percentage)
PDMS	Polydimethylsiloxane
CAR	Carboxen
NIST	National Institute of Standards and Technology
RI	Retention Index
RT	Retention Time
PCA	Principal Component Analysis
Spp.	Species
DB-WAX	Durabond Wax (polyethylene glycol–based stationary phase gas chromatography column)
